# Vaccine-Induced Subacute Thyroiditis (De Quervain’s) after mRNA Vaccine against SARS-CoV-2: A Case Report and Systematic Review

**DOI:** 10.3390/idr14010018

**Published:** 2022-02-21

**Authors:** Giuseppe Pipitone, Lorenzo Vittorio Rindi, Nicola Petrosillo, Nunzio Adalberto Maria Foti, Grazia Caci, Chiara Iaria, Davide Roberto Donno, Evangelo Boumis, Giuseppe Paviglianiti, Fabrizio Taglietti

**Affiliations:** 1 Clinical and Research Department on Infectious Diseases, National Institute for Infectious Diseases “L. Spallanzani”, Via Portuense 292, 00147 Rome, Italy; pep2pe@gmail.com (G.P.); davideroberto.donno@inmi.it (D.R.D.); evangelo.boumis@inmi.it (E.B.); fabrizio.taglietti@inmi.it (F.T.); 2 Department of Medicine, Infectious Disease Unit, ARNAS Civico Di Cristina Hospital, Piazza Leotta Nicola 4, 00128 Palermo, Italy; iaria.chiara@gmail.com; 3 Department of Systems Medicine, Infectious Disease Clinic, Tor Vergata University, Via Montpellier 1, 00133 Rome, Italy; nunziofoti87@gmail.com; 4 Infection Prevention & Control and Infectious Disease Unit, University Hospital “Campus Bio-Medico”, Via Álvaro del Portillo 200, 00128 Rome, Italy; n.petrosillo@unicampus.it; 5 Unit of Infectious Diseases, Department of Clinical and Experimental Medicine, University of Messina, Piazza Pugliatti 1, 98100 Messina, Italy; grazia.caci15@gmail.com; 6 Unit of Paediatric Radiology, ARNAS Civico-Di Cristina Hospital, Piazza Leotta 5, 90100 Palermo, Italy; gpavigl@libero.it

**Keywords:** SARS-CoV-2 vaccine, thyroiditis, De Quervain, ASIA syndrome

## Abstract

De Quervain’s thyroiditis, sometimes referred to as subacute thyroiditis (SAT), is the most common granulomatous disease of the thyroid, typically found after a viral infection in middle-aged women. The mRNA encoding for the angiotensin-converting enzyme-2 (ACE-2) receptor is expressed in follicular thyroid cells, making them a potential target for severe acute respiratory syndrome coronavirus 2 (SARS-CoV-2). Besides infection, SARS-CoV-2 vaccines have also been implicated in SAT pathogenesis. We present a case of a woman developing SAT following vaccination with Comirnaty by Pfizer Inc. (New-York, USA). We performed a systematic review of similar cases available in the literature to provide a better understanding of the topic. We searched the databases PubMed and Embase and followed the Preferred Reporting Items for Systematic Reviews and Meta-Analyses (PRISMA) statement. Patient records were then sorted according to the type of administered vaccine and a statistical analysis of the extracted data was performed. No statistically significant difference between mRNA vaccines and other vaccines in inducing SAT was found, nor was any found in terms of patient demographics, symptoms at presentation, initial, or follow-up blood tests. In our case report, we described the possible association between SARS-CoV-2 mRNA-based vaccine Comirnaty and SAT.

## 1. Introduction

Coronavirus-2019 disease (COVID-19) is a syndrome caused by the RNA virus severe acute respiratory syndrome coronavirus 2 (SARS-CoV-2), the etiological agent of infection in humans. The disease is characterized by respiratory and systemic symptoms, ranging from their complete absence thereof to acute respiratory distress syndrome (ARDS) and acute respiratory failure. The disease is mainly characterized by respiratory, central nervous system (CNS), and gastrointestinal (GI) symptoms. Worldwide diffusion of the virus started in December 2019 and shortly after became an overt pandemic [1]. To date, 265 million cases and 5.2 million deaths have been reported by the WHO [2]. Long-term consequences of SARS-CoV-2 infection have not exactly been defined. One of the largest studies published on post-COVID-19 patients enrolled 1733 patients, out of which 1265 reported at least one symptom among fatigue, sleep disturbances, or muscle weakness [1]. New-onset diabetes was reported to be the main metabolic disorder in these patients, together with disorders of blood coagulation, such as deep vein thrombosis, and of renal function [1]. A few case reports show the association between SARS-CoV-2 infection and thyroid pathologies such as subacute thyroiditis (SAT) [3,4]. The latter, also referred to as De Quervain’s thyroiditis, is the most common granulomatous disease of the thyroid and is typically found after a viral infection in middle-aged women. Disease course is benign, and the pathology is usually self-limited [5]. Signs and symptoms include cervical pain and increased inflammatory markers with onset usually two to eight weeks after a viral upper respiratory tract infection. Laboratory findings may include an elevation in ESR, fT3, fT4; low or absent TSH, anti-TPO and anti-TG titers together with a mild leukocytosis [6]. Furthermore, patients complain of dysphagia, low-to-moderate fever, and symptoms of a general hyperthyroid state [6,7,8]. After an initial hyperthyroid state inducing TSH suppression, a rebound hypothyroid state can be observed before patient regression to euthyroidism [9]. Thyroid ultrasonography during the course of SAT typically shows the gland as globally enlarged, with relative sparing of the isthmus. The thyroid may be diffusely and dishomogeneously hypoechoic or with hypoechoic pseudo-nodular areas with ill-defined borders and of variable dimensions. Said focal elements may show a migrant behavior and frequently relapse during the course of the disease. A Doppler-US study of the pseudo-nodules may reveal hypo-vascularity, a sign of interstitial edema. Vascular signals are often reduced, but if present, these are not interrupted or modified at lesion margins. This could help in differentiating inflammatory pseudo-nodules from nodules. Furthermore, cervical lymphadenopathy is typically found, with involved lymph nodes appearing as enlarged and reactive in nature. A scintigraphic study of SAT revealed an obvious reduction in radiotracer uptake, as a result of organ damage. This finding is common to acute thyroiditis and to Riedel’s chronic thyroiditis [10,11]. Thyroid histology of the early phase of the disease shows interfollicular inflammation with follicles surrounded by T lymphocytes, interstitial infiltrate, interfollicular fibrosis, and non-caseating granulomatous lesions in which most of the cellularity is represented by giant cells, epithelioid histiocytes, and lymphocytes [8,12]. Treatment is based on NSAIDs for pain, glucocorticoids, and β-blockers for the thyrotoxic phase and L-T4 for the hypothyroid phase [7]. Several pathogens have been implicated in its pathogenesis, such as mumps virus, influenza virus, Epstein-Barr virus, coxsackievirus, adenovirus, echovirus, etc. [5]. A genetic correlation with human leukocyte antigen B-35 (HLA-B35) was also described [9]. Before COVID-19, a few studies reported abnormalities in thyroid function and alterations in its follicular architecture in patients with SARS-CoV infection [13]. The pathophysiology of subacute thyroiditis secondary to SARS-CoV-2 infection is thought to be similar to that associated with other viral illnesses, i.e., a result of either a direct infection or of a post-viral inflammatory reaction targeting the thyroid, most frequently in genetically susceptible individuals. Some authors suggest that the affinity of SARS-CoV-2 to the thyroid gland is mediated by angiotensin-converting enzyme 2 (ACE-2) receptors [14]. The mRNA encoding for the ACE-2 receptor is expressed in follicular thyroid cells, making them a potential target for SARS-CoV-2 [15]. Besides SARS-CoV-2 productive infection, its vaccine has also been implicated in the pathogenesis of De Quervain’s thyroiditis. This syndrome was sometimes referred to as autoimmune/inflammatory syndrome induced by adjuvants (ASIA syndrome) [14]. Recent studies described cases of patients presenting with subacute thyroiditis after the inoculation of various types of vaccines against SARS-CoV-2, both inactivated, recombinant or mRNA based [16,17,18,19,20,21,22]. Here, we present a case of a woman developing De Quervain’s thyroiditis following the first dose of mRNA vaccine against SARS-CoV-2 “Comirnaty” by Pfizer Inc. (New-York, USA). We completed our study with a systematic review of similar cases available in the literature in order to provide a better understanding of the topic.

**Figure 1:**
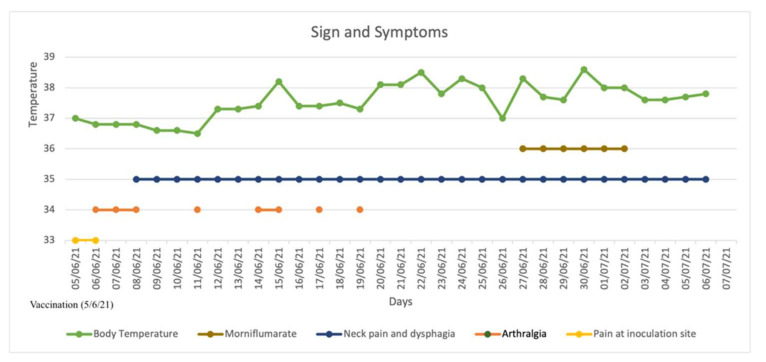
Signs and symptoms from the day of vaccination (5/6/21); patient self-prescribed morniflumate 350 mg/day. Fever and neck pain disappeared on the 2nd day after hospitalization. Patient was hospitalized on 5/7/21 and discharged on 10/07/21.

CASE REPORT: A 49-year-old female patient presented at our hospital complaining of anterior cervical pain, anxiety, dysphagia, fatigue, increased bowel motility, insomnia, night sweats, and palpitations lasting for several days. At admission, EKG showed sinus tachycardia (heart rate: 115 bpm) incomplete right branch block and left-anterior fascicular block; axillary body temperature was 37.3 °C (99.1 °F). Past medical history was negative for thyroid or any other pathology. Family history was negative for autoimmune or thyroid diseases. She was not under any pharmacological therapy, with the exception of a self-prescribed 6-day course of morniflumate, with no reported beneficial effect. Patient denied present or past use of amiodarone, lithium, thyroid replacement hormones, herbal remedies, oral supplements, or drugs of abuse. She denied contracting any infectious disease or traveling during the 2 months prior to hospital admission. She denied being a pet owner or having been in contact with any animal recently. Neck pain and fever disappeared on day 2 after hospitalization. A more detailed history was taken, revealing the anterior cervical pain started 27 days prior to admission and worsened upon swallowing or palpation. Furthermore, the patient reported an intermittent, low-grade fever (fastigium 38.6 °C) with onset during the evening and lasting for 8–12 h every day, starting 21 days before admission, together with night sweats and insomnia. During the following days, her bowel movements increased from twice a week to twice a day, together with a reported weight loss of about 8% of baseline total weight, i.e., from 50 kg to 45 kg, since the beginning of the symptomatology. The patient reported she was vaccinated with the first dose of mRNA vaccine for SARS-CoV-2 (“Comirnaty”—Pfizer) approximately 1 week before the onset of symptoms. For completeness, the patient also complained of fever, mild arthralgia, mild pain at the site of injection fatigue, and headache, approximately 12 h after vaccine inoculation, which self-resolved a few days later. Nevertheless, the patient started keeping a journal for tracking the evolution of body temperature, and any additional signs and symptoms (Figure 1). 

**Figure 2:**
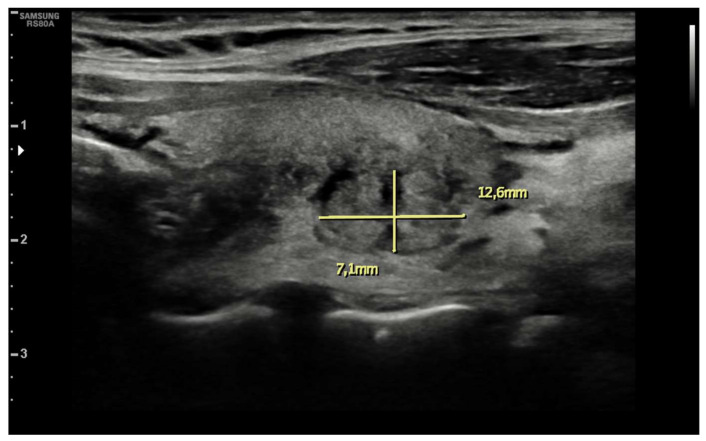
Pseudonodular element in left lobe.

**Figure 3:**
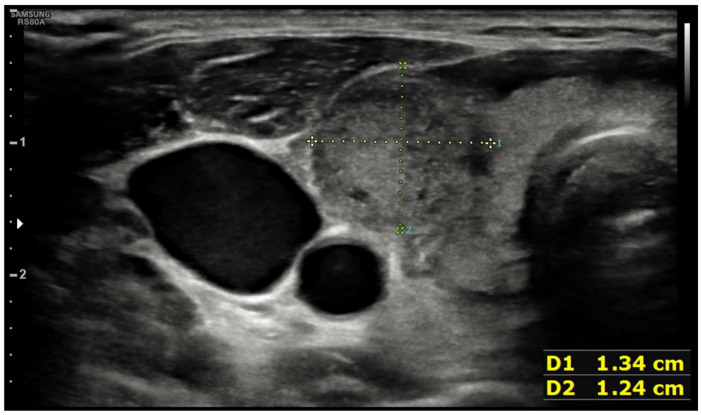
Pseudonodular element in right lobe.

Ten days after vaccine inoculation, the patient performed a nasal swab for SARS-CoV-2 and a second one, namely a multiplex-PCR for respiratory pathogens, both of which resulted in negative. On the next day, the patient performed blood tests, which showed a systemic inflammatory response; White blood cell count (WBC) was 10,800 cells/µL (8200 neutrophils; 1650 lymphocytes), hemoglobin 11.2 g/dL, platelet count 455,000 cells/µL, ESR 88 mm/h, and C-reactive protein (CRP) 75 mg/L. Serum protein electrophoresis showed a non-specific inflammatory picture, with a mild increase in gamma and alpha-2 globulins). ELISA test for EBV, Toxoplasma, CMV, showed an isolated positivity for CMV IgG, which were considered to be non-specific. During the hospitalization, while waiting for blood test results and for an endocrinological consult, patient tachycardia was managed with propranolol 40 mg tris in die (t.i.d.). Blood tests performed in-hospital showed: CRP 78 mg/L, ESR 86 mm/h, Hb 8.7 g/dL, no alteration in WBC or RBC number, no alteration in hepatic or renal function. An abdominal ultrasound showed no pathological findings. A thyroid morphological evaluation was performed via ultrasonography. An appearance typically seen in chronic or sub-acute thyroiditis was observed. Thyroid was increased in dimensions with lobular borders and a micronodular pattern, i.e., hypoechoic nodules with hyperechoic fibrotic shoots. More specifically, two pseudo-nodules were found, the biggest on the left lobe (7 mm × 12.6 mm—Figure 2), and on the right lobe (12 mm × 13 mm—Figure 3).

**Figure 4:**
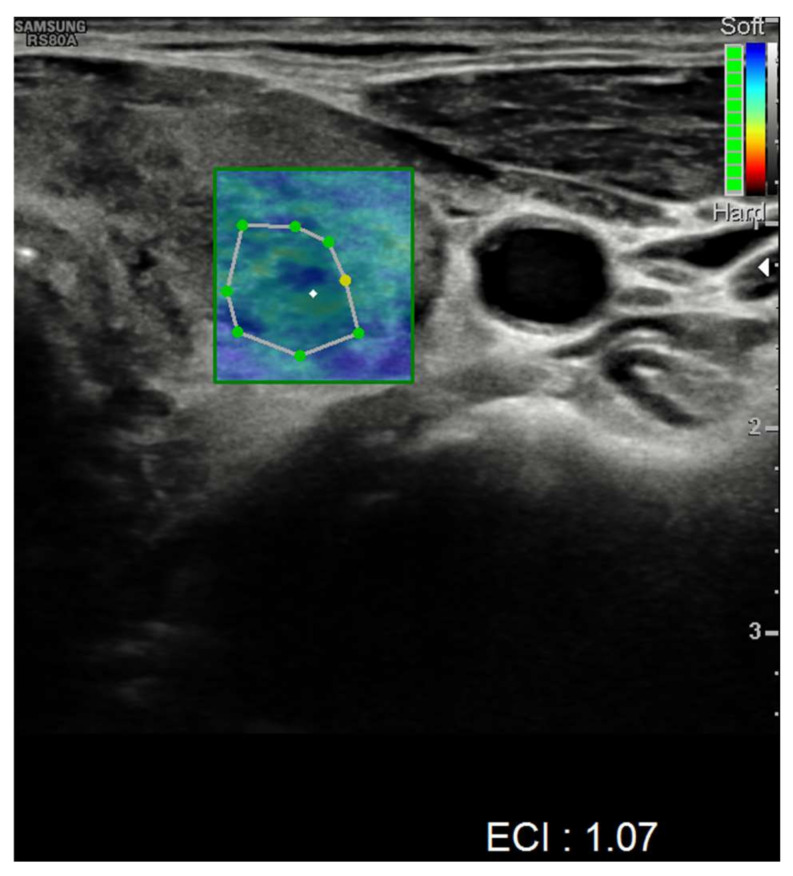
Elasticity contrast index on a nodule in the left thyroid lobe.

**Figure 5:**
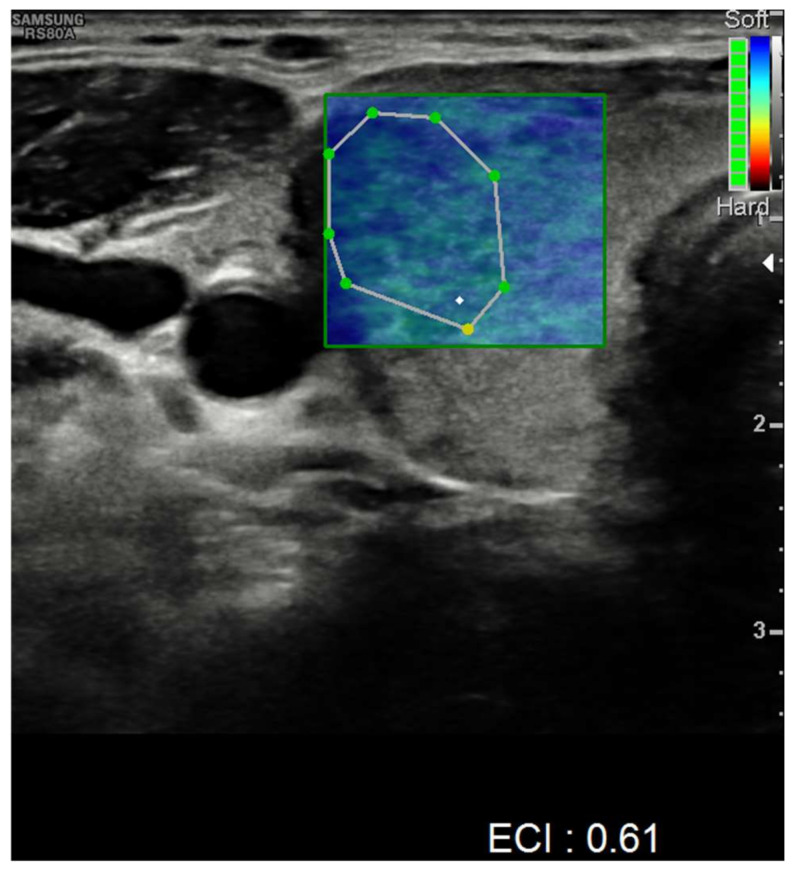
Elasticity contrast index on a nodule in the right thyroid lobe.

Nevertheless, nodules showed no alteration in color-Doppler sampling or in stiffness analysis. The elasticity contrast index (ECI) was computed, resulting in low values. This is a finding compatible with non-malignant nodules (Figure 4 and Figure 5). 

Hormonal tests showed complete thyrotropin suppression with TSH levels < 0.008 microU/mL (normal range 0.4–3.5), elevation of free-T3 12.9 pg/mL (normal range 2.3–4.2), and of free-T4 5.74 ng/dL (normal range 0.8–1.6), antibody titers resulted in negative for thyroglobulin 0.2 U/mL (normal value < 4.5), thyroid peroxidase < 28 U/mL (normal value < 60) and TSH receptor autoantibodies < 1.8 U/L (normal value < 1.8). A full panel for infectious disease serology was requested: Quantiferon (TB-GOLD Plus) was negative, as well as blood cultures (2 sets, sampled 30 min apart). ELISA tests for HIV, HBV, HCV, Parvovirus were negative; CMV IgG was positive, but CMV IgM resulted in negative; EBV VCA IgG and EBV VCA EBNA IgG were positive 167 U/mL (positive value > 20) and 372 U/mL (positive value > 20), respectively. EBV EA IgG, EBV VCA IgM, EBV EBNA IgM were negative; all findings were considered to be non-specific. Widal-Wright resulted in negative as well; nasal swab was repeated at admission and resulted in negative for SARS-CoV-2, as well as SARS-CoV-2-specific serology (Abbott Architect, Immune-enzymatic method): IgG 4.6 (positive > 1.1), IgG for the spike protein receptor-binding domain 666.8 BAU/mL (positive > 7.1), IgM 0.0 (positive > 1.1), and IgA 0.3 (positive > 1.1). Autoimmunity screening panel showed negative results for: Rheumatoid factor, ANA, ENA, anti-ds-DNA, ASMA, AMA, LKM, ANCA, anti-Cardiolipin, anti-b2-microglobulin. An HLA typing was requested, finding * 35 and * 55 in the HLA-B locus, confirming the important role of the genetic background of the disease [9]. The clinical picture and laboratory findings were consistent for subacute (De Quervain’s) thyroiditis. In our opinion, given the characteristics of the symptomatology, the timing of presentation onset, and excluding all other possible causes, the only factor that could have caused the pathology was the inflammatory response to the vaccine. For completeness, during our first evaluation, TSH suppression was interpreted as potentially caused by primary hyperthyroidism or by an “autonomous” thyroid nodule. For this reason, the patient was initially started on methimazole (5 mg tid). Nevertheless, after a better understanding of the case, and after a thyroid ultrasound was obtained, treatment was switched to prednisone, NSAIDs, and beta-blockers, and then gradually tapered to its interruption over the next month. The follow-up blood test examination at 1 week and 3 weeks showed a reduction in inflammatory markers and normalization of thyroid hormones. At one week after discharge, lab tests showed: ESR 53 mm/h, TSH 0.008 mUI/mL, fT3 3.49 pg/mL, fT4 1.6 ng/mL. At week 3 after discharge, the following lab tests were obtained: ESR 25 mm/h, TSH 0.208 mUI/mL, fT3 1.52 pg/mL, fT4 0.73 ng/mL.

## 2. Materials and Methods

### Systematic Review of the Literature

Three independent researchers (LVR, GP, and NAF) performed a systematic search of the literature via the databases Pubmed and Embase. The searched terms for both databases were [“COVID-19” and “vaccine” and “subacute thyroiditis”] and [“COVID-19” and “vaccine” and “De Quervain”]. The search period was from 1 January 2020 to 14 September 21. Records were then sought for retrieval and assessed for validity and relevance, and subsequently entered into a computerized database. We considered relevant all previous studies reporting subacute thyroiditis following any type of SARS-CoV-2 vaccination regardless of the study design. Preprint or inaccessible articles were not included. We did not restrict our search to any Country or language. We conducted our systematic review according to the Preferred Reporting Items for Systematic Reviews and Meta-Analyses (PRISMA) statement [23]. Patient records were then sorted according to the type of administered vaccine, i.e., mRNA vaccines (Comirnaty and Spikevax), inactivated virus (CoronaVac), or vector vaccine (ChAdOx1). Statistical analysis was performed using SPSS Statistics (v. 27). Population characteristics, including age, sex, pharmacological treatment, ultrasonographic findings, symptoms at presentation, etc. were gathered and calculated as number (%). For these categorical data, we used a contingency table with the Fisher test (a < 0.005). Blood test results are shown as median and interquartile range (25–75%). For quantitative variables, we used the Mann–Whitney U test (a < 0.005, confidence interval 95%). We did not include the case reported by Patel KR et al. in our statistical analysis, since the specifics about the type of vaccine administered to the patient were not described in the study.

## 3. Results

**Figure 6:**
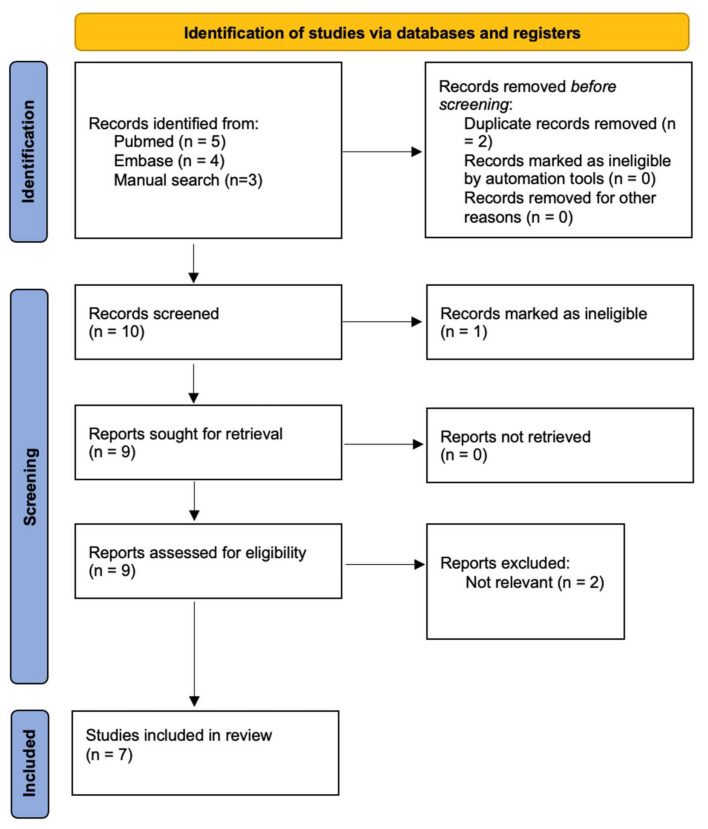
Study retrieval and selection process.

A total of 9 studies were found through systematic database searching and 3 via a manual search of referenced articles. Duplicate records were removed, and the remaining 10 studies were retrieved and assessed for relevance and validity, leaving 7 studies for inclusion. A total of 10 cases were described. Records retrieval and selection process can be found in Figure 6.

Retrieved relevant information can be found in Table 1, while population characteristics including age, sex, pharmacological treatment, ultrasonographic findings, symptoms at presentation can be found in Table 2.

All reported cases described De Quervain thyroiditis induced by SARS-CoV-2 vaccination: 4 cases secondarily to an mRNA vaccine inoculation, 6 caused by inoculation of a viral vectorial vaccine or inactivated viral vaccine, and 1 secondarily to an unspecified SARS-CoV-2 vaccine. In our analysis, no statistically significant difference between mRNA vaccines (4 cases: 2 Comirnaty and 2 Spikevax) and other vaccines (6 cases: 4 CoronaVac and 2 ChAdOx1) in inducing SAT was found in terms of patient demographic characteristics such as age or sex, symptoms at presentation, initial or follow-up blood tests. In one case, the author did not specify the type of vaccine that was employed and therefore his study was excluded from the statistical analysis. All the SAT cases that developed secondarily to SARS-CoV-2 vaccination reported in our review are characterized by the absence of thyroid autoantibodies. All described patients showed a suppressed TSH at presentation, with normalization of TSH values taking place over 30 days or more. In one case, the reported patient showed positivity for anti-TG antibodies, and therefore TSH was higher than the upper limit even at 6 weeks after the initial blood test [20]. Only in one case was the patient treated with levothyroxine [20]. Thyroid echography showed no difference according to the type of vaccine used: one or more hypoechoic areas (81.8%), half of which were reported to be characterized by a heterogeneous echotexture (45.5%) and a reduced blood flow at doppler US (54.5%).

## 4. Discussion

Subacute thyroiditis is caused by a T-cell-mediated hypersensitivity to viral antigens found in infected epithelial cells [8]. Diagnosis is usually performed by clinical features, laboratory findings, and ultrasound, whereas fine needle aspiration is reserved for selected cases to differentiate it from a thyroid neoplasm [5]. SARS-CoV-2 infection can act as a trigger for subacute thyroiditis [24], and a few cases of subacute thyroiditis were also reported after the SARS-CoV-2 vaccine [16,17,18,19,20,21,22], also referred to as ASIA syndrome [16]. Autoimmune/inflammatory syndrome induced by adjuvants (ASIA) is responsible for numerous autoimmune disorders such as systemic lupus erythematosus, rheumatoid arthritis, or autoimmune endocrinopathies such as Hashimoto and subacute thyroiditis. ASIA syndrome was first described in 2011 by Shoenfeld and Agmon-Levin, followed by studies reporting a correlation to the presence of specific class II HLA alleles [25]. Adjuvants are substances capable of triggering or enhancing autoimmunity via various pathways, e.g., B lymphocyte activation. Among these, aluminum and silicon are the most frequently used. Aluminum is present as an adjuvant in various vaccines, such as the one against HBV, influenza, and HPV. Furthermore, several studies report subacute thyroiditis following the seasonal flu vaccine [25]. We described in our case report the possible association between mRNA-based vaccine Comirnaty by Pfizer and subacute (De Quervain’s) thyroiditis. Our review and statistical analysis showed no relevant difference in population characteristics between patients undergoing mRNA or vector-based SARS-CoV-2 vaccination in inducing SAT. Comirnaty immunogenicity is based on an mRNA coding for the S protein of SARS-CoV-2. The S protein contains a cleavage site specific for furin. Furin proteases render the virus more aggressive and pathogenic with respect to the H1N1 virus, which shows a trypsin protease-mediated cleavage system instead. The mRNA contained in the vaccine is protected by a four-lipid compound. In this vaccine, no type of adjuvant is employed, since mRNA itself acts as adjuvant [26,27].

## 5. Conclusions

As a result of shared efforts in population-based vaccination against SARS-CoV-2, many people have now undergone either SARS-CoV-2 infection or passive immunization. Therefore, subacute thyroiditis may be the result of any already known factor, coincidentally superimposed to either infection or vaccination. Nevertheless, we cannot exclude the causal link. For this reason, clinicians should consider the possibility of subacute thyroiditis after SARS-CoV-2 antigenic challenge, secondarily to immunization or infection, in order to treat patients accordingly. No statistically relevant conclusion can be drawn from our analysis, due to the limited cases found in the literature and to the heterogenicity of the described affected population. 

On a final note, the reported case and literature review should not justify a negative attitude against vaccination, which remains one of the most efficient public health strategies against the ongoing pandemic. 

## Figures and Tables

**Table 1:** Brief overview of all available similar cases, relevant information, and test results.

	Oyibo			Franquemont and Galvez		Şahin Tekin et al.		İremli et al.						Bornemann et al.			
	At Presentation	At 6 Weeks	At 12 Weeks	At Presentation	At 3 Weeks	At Presentation	At 9 Weeks	1 at Presentation	1 at 4 Weeks	2 at Presentation	2 at 4 Weeks	3 at Presentation	3 at 4 Weeks	1 at Presentation	1 At 6 Weeks	2 at Presentation	2 at 4 Weeks
Gender	Female			Female		Male		Female		Female		Female		Female		Female	
Age	55			42		67		35		34		37		26		49	
Vaccine	(I dose) ChAdOx1^®^, AstraZeneca plc (Cambridge, UK)			(I dose) Comirnaty, Pfizer		(II doses) CoronaVac^®^, Sinovac Biotech (Beijing, China)		(II doses) CoronaVac^®^, Sinovac		(I dose-prev. infection) CoronaVac^®^, Sinovac		(II doses) CoronaVac^®^, Sinovac		(I dose) ChAdOx1, AstraZeneca^®^		(I dose) Spikevax^®,^ Moderna inc. (Massachusetts, USA)	
Symptom onset from vaccination (days)	21 days			5 days		19 days (from II dose)		5 days (from second dose)		4 days		7 days (from II dose)		14 days		14 days	
Signs and symptoms at presentation	Neck pain and swelling, headache, palpitations, sore throat, generalized aches.			Sore throat, palpitations, sinus tachycardia on EKG (>130 bpm)		Fever, hypertension, weight loss (−4 kg in less than 30 days), left ear and anterior neck pain and tenderness at palpation		Severe neck pain, palpitations, fever, fatigue. Thyroid tenderness and enlargement		Anterior neck pain, fatigue, weight loss, fever, palpitations		Anterior neck pain and tenderness.		Cervical pain, fever, chills, thyroid tenderness, cervical lymphadenopathy		Headaches, difficulty concentrating, cervical sore throat, thyroid tenderness	
TSH mU/L (r.v. 0.3–4.2)	0.09	20.3	5.35	<0.01	<0.01	<0.005	3.15	0.47	2.27	0.01	<0.015	0.9	0.018	1.75	0.83	0.05	0.01
fT3 ng/mL (r.v. 2.7–4.3)	NA	NA	4.2	7.68	8.78	8.06	2.94	4.01	3.45	7.68	5.22	3.94	4.55	3.72	2.6	3.25	3.97
fT4 pg/mL (r.v. 0.93–1.7)	1.96	4.7	1.21	4.58	3.2	2.87	0.97	1.09	1.49	0.4	2	1.07	2.03	0.72	0.69	0.73	1.08
Anti-TPO IU/mL (r.v.)	<10 (r.v. < 34)	NA	NA	<28	NA	Neg	NA	1.2 (0–9)	NA	1.2	NA	4.1	NA	Neg	Neg	Neg	33 (<6)
Anti-TG IU/mL (r.v.)	NA	NA	15 (r.v. < 3)	NA	Neg	Neg	NA	<0.9	NA	<0.9	NA	<0.9	NA	Neg	Neg	Neg	Neg
TRAB IU/L (r.v.)	NA	NA	1.9 (<2.9)	NA	Neg	Neg	NA	<1.5	NA	<1.5	NA	<1.5	NA	Neg	Neg	Neg	Neg
ESR (mm/hr)	51	NA	NA	62	26	67	4	53	28	19	16	25	44	NA	NA	NA	NA
CRP (mg/L)	87	NA	NA	NA	Normal	53.9	3.6	100.5	13.1	6	5.3	2.4	NA	29.4	1.0	21.9	22.4
WBC (10^9^^/^L)	8.5	NA	NA	NA	NA	NA	NA	9.9	11.1	9.7	9.7	6.3	7.8	14.3	9.77	7.86	5.75
PLT (10^9^^/^L)	491	NA	NA	NA	NA	NA	NA	NA	NA	NA	NA	NA	NA	NA	NA	NA	NA
HLA-B35	NA			NA		NA		NA		NA		NA		NA		NA	
Ultrasound findings at presentation	Enlarged thyroid gland with heterogeneous echotexture. No nodules no hypervascularity; reduced vascular flow to right lobe			NA		Reduced echogenicity and diffusely heterogeneous texture with pseudonodular areas consistent with thyroiditis		Bilateral focal hypoechoic areas with decreased blood flow on Doppler US		Bilateral focal hypoechoic areas with decreased blood flow on Doppler US		Numerous hypoechoic areas with decreased blood flow on Doppler US		Heterogeneous echogenicity and bilateral hypoechoic areas with decreased blood flow on Doppler US		Hypoechoic confluent areas with decreased vascularity on Doppler US	
Therapy	Propranolol, ibuprofen, paracetamol	Levothyroxine	Levothyroxine	Prednisone, propranolol	NA	Ibuprofen	-	Methylprednisolone, propranolol	methylprednisolone	Methylprednisolone, propranolol	methylprednisolone	No treatment	No treatment	Ibuprofen, prednisolone	-	Diclofenac	Prednisolone
	**Patel M. et al.**					**Patel KR. et al.**						Pipitone et al.					
	**At presentation**			**At 4 weeks**		**At presentation**			**At <8 weeks**			**At presentation**			**At 21 days**		
Gender	Female					Male						Female					
Age	46					48						49					
Vaccine	(II doses) Spikevax, Moderna^®^					(II doses) Unspecified SARS-CoV-2 vaccine						(I dose) Comirnaty, Pfizer^®^					
Symptom onset from vaccination (days)	32 days from I dose (right following II dose)					7 days after II dose						7 days					
Signs and symptoms at presentation	Neck pain, tender, warm, swollen, and firm area on right thyroid. Tachycardia (119 bpm), hypertension (146/76 mmHg).					Right neck swelling, throat discomfort, palpitations, fever, weight loss (−4.5 kg). Tender fullness on right anterior neck.						Anterior cervical pain, anxiety, dysphagia, fatigue, increased bowel movements, insomnia, night sweats, palpitations, tachycardia (115 bpm), fever (38.6 °C), weight loss (−8%)					
TSH mU/L	0.00			0.365		0.01			Suppressed			<0.008			0.208		
fT3 pg/mL (r.v.)	Elevated			2.96		NA			NA			12.9 (2.3–4.2)			1.52		
fT4 ng/dL (r.v.)	Elevated			1.02		3.6			decreased			5.74			0.73		
Anti-TPO IU/mL (r.v.)	Negative			NA		NA			NA			<28 (<60)			NA		
Anti-TG IU/mL (r.v.)	Negative			NA		NA			NA			0.2 (<4.5)			NA		
TRAB IU/L (r.v.)	NA			NA		NA			NA			<1.8 (<1.8)			NA		
ESR (mm/hr)	49			NA		Elevated			Decreased			86			25		
CRP (mg/L)	86			NA		Elevated			Decreased			78			NA		
WBC (10^9^^/^L)	NA			NA		NA			NA			10,8			NA		
PLT (10^9^/L)	NA			NA		NA			NA			455			NA		
HLA-B35	NA					NA						* 35, * 55					
Ultrasound findings at presentation	Asymmetric thyroid enlargement with two hypoechoic areas with no internal vascularity on right thyroid lobe					Diffuse thyroid enlargement with hypoechoic areas with heterogeneous echotexture and no evidence of increased vascularity						Diffuse enlargement of thyroid gland with hypoechoic nodules with hyperechoic shoots (micronodular pattern) without increase in vascularity					
Therapy	Analgesics, dexamethasone, propranolol			-		Prednisolone and NSAIDs			-			Prednisolone, propranolol			-		

**Table 2:** Population characteristics are shown in the table. Age, sex, pharmacological treatment, ultrasonographic findings, symptoms at presentation, SAT after I dose are shown as number (%). Blood tests are shown as median and Interquartile range (25–75%—confidence interval 95%). Patients are divided according to type of administered vaccine: “mRNA vaccine (Pfizer and Moderna)”, “inactivated virus (Sinovac), or vector vaccine (Astrazeneca)”. Palpitations and tachycardia were reported together as non-rhythmic cardiac alterations. SAT = subacute thyroiditis; TSH = thyroid-stimulating hormone; fT3 = free triiodothyronine; fT4 = free thyroxine; ESR = erythrocyte sedimentation rate; CRP = C-reactive protein; WBC = white blood cells; NSAIDs = nonsteroidal anti-inflammatory drugs; T0 = time at presentation; T4 = follow-up at >3–4 months. Data are shown in number and (%).

	Overall (*n* = 10)	mRNA Vaccines (*n* = 4)	Inactivated/Vector Vaccines (*n* = 6)	*p*-Value
Female	9 (90%)	4 (100%)	5 (83.3%)	1
SAT after I dose	6 (60%)	3 (75%)	3 (50%)	0.571
Days between I/II dose and symptoms onset	10.5 (5–19.5)	10.5 (5.5–27.5)	10.5 (4.75–19.5)	0.762
Age (years)	44 (34.75–50.50)	47.5 (43–49)	36 (32–58)	0.476
Symptoms				
Neck pain	9 (90%)	3 (75%)	6 (100%)	0.400
Sore Throat	4 (40%)	3 (75%)	1 (16.7%)	0.191
Palpitation/Tachycardia	6 (60%)	3 (75%)	3 (50%)	0.571
Headache	3 (30%)	2 (50%)	1 (16.7%)	0.333
Fever	5 (50%)	1 (25%)	4 (66.7%)	0.262
Fatigue	3 (30%)	1 (25%)	2 (33.3%)	0.667
Weight loss	3 (30%)	1 (25%)	2 (33.3%)	1
Hypertension	3 (30%)	2 (50%)	1 (16.7%)	0.333
Anxiety	1 (10%)	1 (25%)	0 (0%)	0.400
Night sweats	1 (10%)	1 (25%)	0 (0%)	0.400
Increase in bowel motility	1 (10%)	1 (25)	0 (0%)	0.400
TSH at T0 (mU/L)	0.03 (0.073–0.578)	0.09 (0.02–0.04)	0.28 (0.08–1.11)	0.114
TSH at T4	0.287 (0.10–2.49)	0.109 (0.01–0.33)	1.55 (0.01–3.70)	0.257
fT3 at T0 (pg/mL)	4.01 (3.49–7.87)	7.68 (3.25-)	3.98 (2.79–7.78)	0.548
fT3 at T4	3.71 (2.86–4.72)	3.47 (1.88–7.58)	3.83 (2.86–4.72)	1
fT4 at T0 (ng/dL)	1.09 (0.73–3.73)	4.58 (0.73-)	1.08 (0.64–2.19)	0.262
fT4 at T4	1.35 (0.91–2.26)	1.05 (0.8–2.67)	1.35 (0.90–2.01)	0.610
ESR at T0 (mm/h)	52 (31–65.75)	62 (49-)	51 (22–60)	0.393
ESR at T4	25.5 (13–32)	25.5 (25-)	22 (7–40)	1
CRP at T0 (mg/L)	53.9 (13.95–86.5)	78 (21.9-)	41.65 (5.1–90.38)	0.905
CRP at T4	5.3 (2.3–17.75)	22.4 (22.4–22.4)	4.45 (1.65–11.15)	0.400
WBC at T0 (cells/mL)	9700 (7860–10,800)	9330 (7860-)	9700 (7400–12，100)	1
WBC at T4	9700 (6775–10,435)	5750 (5750–5750)	9735 (8275–10，770)	0.400
Pharmacological treatment				
NSAIDs	4 (40%)	1 (25%)	3 (50%)	0.571
Steroids	7 (70%)	4 (100%)	3 (50%)	0.200
Beta-blockers	5 (50%)	2 (50%)	3 (50%)	0.738
Methimazole	1 (10%)	1 (25%)	0 (0%)	0.400
Levothyroxine	1 (10%)	0 (0%)	1 (16.7%)	0.600
Ultrasonographic findings				
Hypoechoic areas	8 (80%)	3 (75%)	5 (83.3%)	1
Heterogeneous echotexture	4 (40%)	1 (25%)	3 (50%)	0.571
Reduced blood flow at Doppler-US	6 (60%)	1 (25%)	5 (83.3%)	0.119
